# In Reference to the Original Article “Three Calcium Hydroxyapatite‐Based Dermal Fillers Marketed in Mexico: Comparison of Particle Size and Shape Using Electron Microscopy”

**DOI:** 10.1111/jocd.70992

**Published:** 2026-06-18

**Authors:** Gilberto Antonio Sanchez Rico

**Affiliations:** ^1^ Oneline Beauty Clinic Cancún Quintana Roo Mexico

Dear Editor,

1

We thank the correspondents for their continued interest in our work and the opportunity to further clarify its scope and intent.

As explicitly stated in the original manuscript, the study was designed as a descriptive and exploratory analysis of calcium hydroxyapatite (CaHA) microsphere morphology using scanning electron microscopy (SEM). It was not intended to establish physicochemical equivalence, comparative performance, or clinical superiority among products. Methodological limitations, including the use of a single syringe per product without batch replication was clearly acknowledged to prevent overinterpretation and overgeneralization [1].

SEM allows morphological characterization only and does not permit prediction of in vivo behavior or clinical outcomes [2]. Accordingly, the discussion contextualized the observed morphological features within the existing biomaterials literature, without asserting mechanistic, inflammatory, or performance‐based conclusions [3]. Differences in formulation components and carrier matrices were recognized as limiting direct surface comparisons and further reinforce the exploratory nature of the observations [1, 4].

Any reference to biocompatibility was framed cautiously and exclusively within a hypothesis‐generating context, consistent with established principles in biomaterials science [2, 3]. No causal relationships or clinical consequences were proposed. The manuscript explicitly emphasizes the need for additional in vivo, histopathological, and immunological studies to better understand how morphological characteristics of CaHA microparticles may influence tissue response [1, 5, 6, 7].

Statements regarding hyaluronic acid filler longevity were included solely as general contextual information and were not intended to imply uniform behavior across products. Variability related to formulation, indication, technique, and patient factors is well documented and was not disputed [8, 9, 10, 11, 12, 13, 14, 15].

The manuscript does not favor, promote, or disadvantage any specific product or manufacturer. All disclosures complied fully with journal policy. Educational speaking activities for multiple companies were transparently declared and were not related to study design, funding, data acquisition, analysis, or interpretation. This study received no industry support, and manufacturers had no involvement at any stage. The authors confirm the accuracy and completeness of the original disclosure.

We trust that these clarifications reinforce the intended scientific framework of the study and preserve a clear distinction between exploratory morphological research and clinical interpretation.

## Conflicts of Interest

The author declares no conflicts of interest.

## Linked Articles

This article is linked to Furman‐Assaf and Korman papers. To view these article, visit https://doi.org/10.1111/jocd.70758.

## Data Availability

Data sharing not applicable to this article as no datasets were generated or analysed during the current study.
